# Supplementary material for: Ethylene Acts as a Local and Systemic Signal to Mediate UV-B-Induced Nitrate Reallocation to Arabidopsis Leaves and Roots via Regulating the ERFs-NRT1.8 Signaling Module
Source: Int J Mol Sci. 2022 Aug 13;23(16):9068. doi: 10.3390/ijms23169068 (PMC9408821; doi:10.3390/ijms23169068)
Supplement: Supplementary file 1 [file ijms-23-09068-s001.zip › ijms-1836730-supplementary.pdf]

Supplementary materials

Table S1. List of primers used in this study

Primer Name	Primer Sequence（5'-3'）
NRT1.8-F	TCTTCATCTTCGCATACAGGCGGT
NRT1.8-R	GCCATTATCGCAATCACAAGCCCA
ORA59-F	AAGGGATAAGAGTGTGGCTTGGGA
ORA59-R	CTTTCAAAGCGAAAGCCGCCTGAT
ERF1B-F	GAGGAAACACTCGATGAGACG
ERF1B-R	GGAGCGGTGATCAAAGTCAC
ERF104-F	GAACCATCACCAACCAATCC
ERF104-R	GTCCCAAGCCAGATCCTACA
ACS2-F	GACATCGCTAATTTCCAAGACTACCA
ACS2-R	TCCTCCGCTCATAACCACCCTCT
ACS4-F	CTTTCTTGCTTCCCACGCCATAT
ACS4-R	TCTTGGCTTGCTCGTAGGCTTCT
ACS5-F	GCTATGGCTGAGTTTATGGAAGA
ACS5-R	ATCCAGGATAGTAAGGAGTAGGC
ACS6-F	CGAGTTTCGGTCTTGTTTCGTCA
ACS6-R	GTGGTTATCTCAGCGTGCCTTGC
ACS7-F	GCTTCACGCTTGTCTCGTCTCAG
ACS7-R	TCCCTTTCAAACACTCAATCCCT
ACS8-F	GGGTGATTTACTCCAACGATGAT
ACS8-R	CTGATAACAATGCGGACAAGAGG
ACS11-F	GCAGGTTTGTTCTGTTGGGTTGA
ACS11-R	CATCGTTTGGTCCGACATATTCG
ACO1-F	AGGAACTCAGCAAGACGATG
ACO1-R	AAGAACTCAAGACCAGGCAC
ACO2-F	CGGGAAGTATAAGAGTGTGCTG

ACO2-R	GGGTACTCGGAATCTTTCTCG
ACO3-F	GATGGTGATTGGGTTGATGTTC
ACO3-R	AGTCCGGCATAGAGTTTCATG
ACO4-F	TTTCTACCTCAAGCACCTTCC
ACO4-R	AACTTCTCTATCTTTCCGGCG
ACO5-F	GGAGGTGTCGTTTTGCTTTTC
ACO5-R	ACTCTTGTACCTTCCGTTGC
ACTIN2-F	CAAGGCCGAGTATGATGAGG
ACTIN2-R	GAAACGCAGACGTAAGTAAAAAC

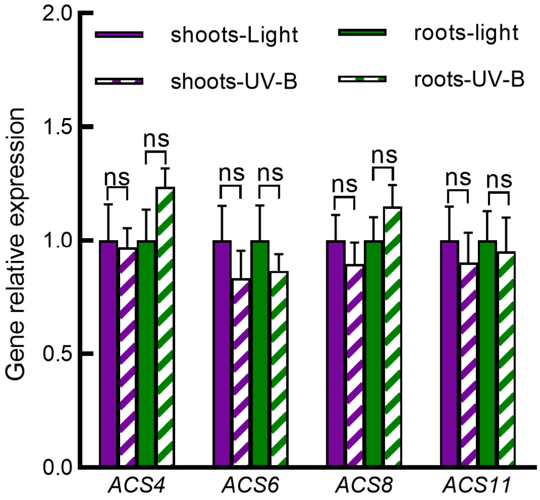

Figure S1. Effect of UV-B on expression of *ACS* genes in wild-type Col-0 shoots and roots. Expression of *ACS* genes were determined by qPCR in shoots and roots of 20-day-old wild-type Col-0 plants exposed to light alone (Light) or with 0.5 W/m^2 UV-B (UV-B) for 3 h. Data are means ± SE of three biological replicates and presented as values relative to those under light treatment after normalization to those of *ACTIN2*. Bars with "ns" indicate no significant difference from those under light treatments.

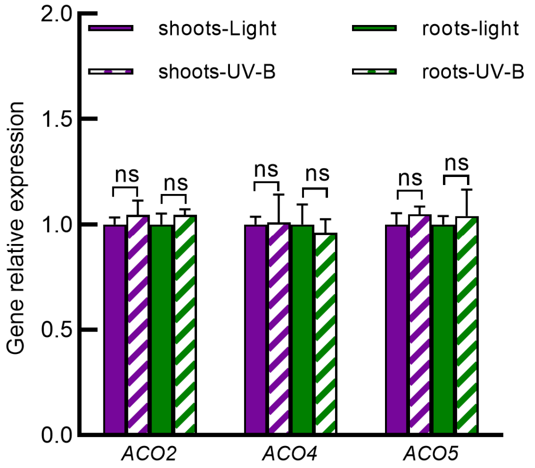

Figure S2. Effect of UV-B on expression of *ACO* genes in wild-type Col-0 shoots and roots. Expression of *ACO* genes were determined by qPCR in shoots and roots of 20-day-old wild-type Col-0 plants exposed to light alone (Light) or with 0.5 W/m^2 UV-B (UV-B) for 3 h. Data are means ± SE of three biological replicates and presented as values relative to those under light treatment after normalization to those of *ACTIN2*. Bars with "ns" indicate no significant difference from those under light treatments.
